# Rhabdomyolysis After Risperidone Overdose in a Patient With Schizophrenia: A Case Report

**DOI:** 10.1002/npr2.70037

**Published:** 2025-07-09

**Authors:** Jun Takahashi, Kota Kikuchi, Rui Inano, Yasushi Kawamata, Norio Sugawara, Norio Yasui‐Furukori

**Affiliations:** ^1^ Department of Psychiatry School of Medicine Dokkyo Medical University Shimotsuga Tochigi Japan

**Keywords:** overdose, rhabdomyolysis, risperidone, schizophrenia, serotonin receptors

## Abstract

A 50‐year‐old woman with schizophrenia overdosed on risperidone, lormetazepam, and lorazepam and subsequently developed rhabdomyolysis, with elevated creatine kinase (CK) and myoglobin levels. Intravenous treatment stabilized her condition. Risperidone was restarted at a regular dose, and rhabdomyolysis did not recur. She was discharged after 29 days, and she continued outpatient care without further complications. There have been no reports of rhabdomyolysis caused by overdosing on risperidone alone. This case is important for understanding the risk profile of this drug.

## Abstract 2

Clinical course of the patient including creatine kinase (CK). After high dose of risperidone, CK spiked, then gradually decreased and reached normal range.

## 1 Introduction

Rhabdomyolysis is a condition characterized by the release of muscle fiber contents into the bloodstream due to the destruction of muscle tissue. Rhabdomyolysis is usually associated with strenuous exercise, ischemia, or the use of certain medications, such as statins and antibiotics; however, it has been confirmed that psychiatric medications, especially antipsychotics, are associated with a high risk of inducing this condition [1, 2].

In general, risperidone rarely causes rhabdomyolysis, but there have been isolated reports of rhabdomyolysis caused by regular doses of risperidone or interactions of risperidone with other drugs [3, 4, 5, 6, 7, 8]. Notable examples include combinations such as risperidone (4 mg) with topiramate (200 mg) [4], risperidone (4 mg) with simvastatin (10 mg) [5], risperidone (6 mg) with cerivastatin (0.2–0.4 mg) [6], risperidone (1 mg) with escitalopram (10 mg) [7], and risperidone (8 mg) with mirtazapine (45 mg) [8]. These cases suggest that drug–drug interactions may play a significant role in the pathogenesis of antipsychotic‐associated rhabdomyolysis, even when standard therapeutic doses are administered.

However, there have been no reports of rhabdomyolysis caused by an overdose of risperidone [3]. In this report, we describe a case of rhabdomyolysis caused by an overdose of risperidone in a patient with a history of schizophrenia. Written consent for this case report was obtained from the patient.

## 2 Case Presentation

A 50‐year‐old woman with a history of schizophrenia presented to the emergency department of our hospital with acute onset of generalized pain and fatigue after taking 75 mg of risperidone, approximately 10 h before being found unconscious, for a suicide attempt. She started hearing voices 1 year prior to this presentation after the death of her sister. She began to believe that she was being bugged through electrical sockets and that an evil organization was trying to kill her. As a result, she was admitted to an area psychiatric hospital and treated with 1 mg of risperidone and 3 mg of lorazepam for short‐term psychosis. Her psychiatric symptoms were rapidly alleviated, and after she was discharged from the hospital, she continued to receive outpatient treatment at the same hospital. In the outpatient clinic, she was treated with 2 mg of risperidone, 0.5 mg of lorazepam, and 5 mg of lormetazepam as needed. Because the hallucinations and delusions persisted, the diagnosis of schizophrenia was confirmed 3 months after the initial presentation, and the patient was informed of the diagnosis. Unfortunately, the patient was unable to accept this diagnosis, and she gradually became less compliant with treatment, with longer intervals between hospital visits. 4 months after the initial presentation, the patient's neighbor visited, but she did not respond. The patient's brother rushed over to the patient's residence and found the patient unconscious. She was then taken to the emergency room of our hospital. Her brother found an empty pack of 75 doses of 1 mg risperidone in the trash.

**FIGURE 1:**
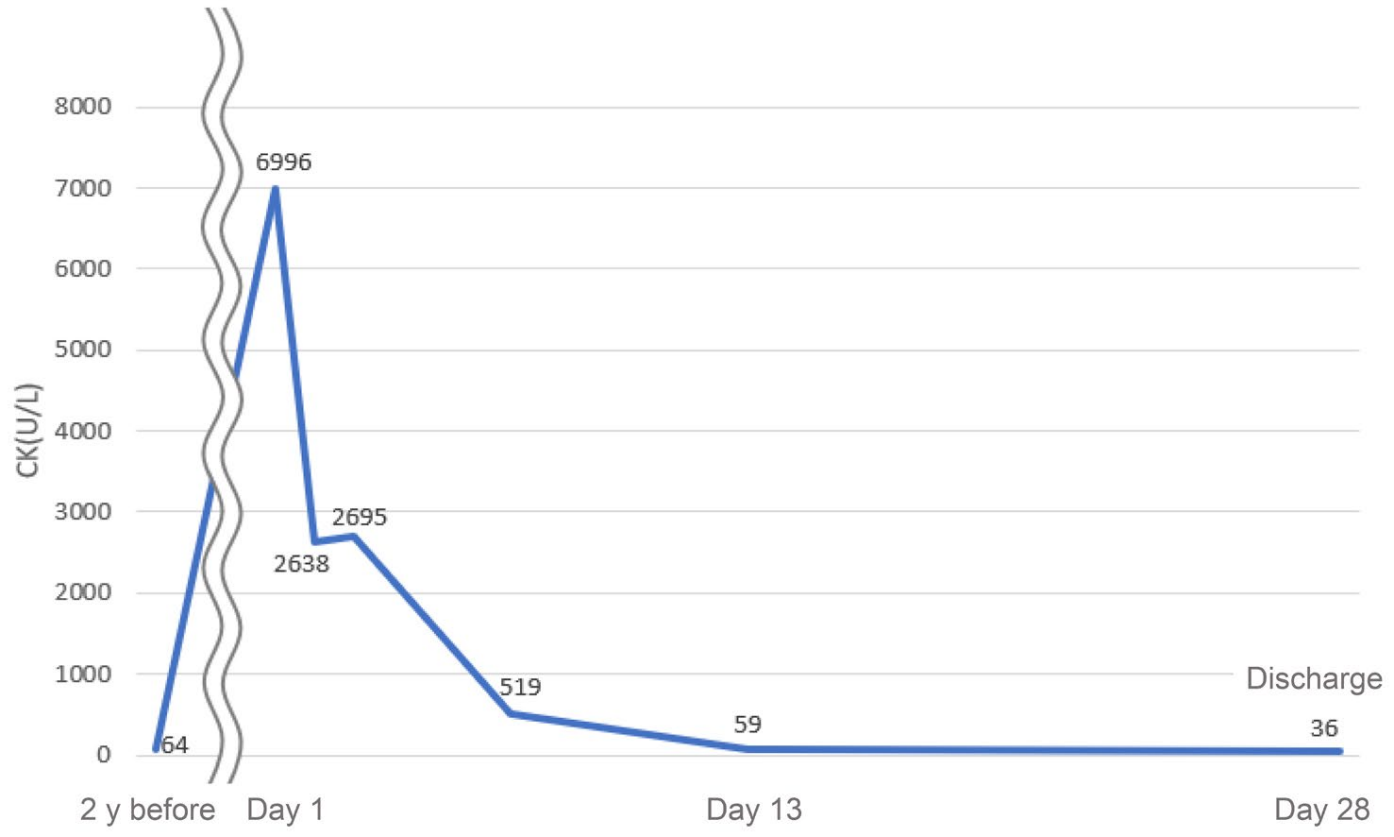
Clinical course of the patient including creatine kinase (CK). After high dose of risperidone, CK spiked, then gradually decreased and reached normal range.

Upon admission, the patient was found to have a flat facial expression, no eye contact, auditory hallucinations, delusions of observation, and delusions of being harmed. In addition, the patient's laboratory tests on admission revealed significantly elevated levels of creatine kinase (CK) (6996 U/L: normal range 41–153 U/L), myoglobin (3728 ng/mL: normal range less than 109 ng/mL), aspartate transaminase (AST) (114 U/L: normal range 13–30 U/L), alanine transaminase (ALT) (47 U/L: normal range 7–23 U/L), and lactate dehydrogenase (LDH) (323 U/L: normal range 124–222 U/L). On admission, the patient's vital signs were stable (blood pressure, 114/78 mmHg; oxygen saturation, 96%; and body temperature, 37.3°C). However, the patient complained of generalized myalgia. To assess the extent of muscle damage and its effect on the kidneys, a diagnostic evaluation was performed, including a renal function test and serum electrolyte measurement, but no abnormalities were found (creatine 0.56 ng/mL: normal range 0.46–0.79 mg/mL, estimated glomerular filtration rate (eGFR) 85.2 mL/min: normal range more than 60 mL/min). X‐ray and electrocardiogram evaluations also revealed no abnormalities. On the basis of the above findings, rhabdomyolysis was diagnosed, and intravenous fluid therapy with isotonic saline was initiated to prevent renal failure. During her hospitalization, her symptoms were alleviated, and her biochemical marker levels gradually reduced. The patient's muscle pain and biochemical marker levels normalized within 2 weeks (Figure 1), but because the hallucinations worsened, 2 mg of risperidone was restarted. However, there was no further increase in CK levels or muscle pain, and with the stabilization of psychiatric symptoms, the patient was discharged 29 days after admission. She continued to take her medications on an outpatient basis. There was no recurrence of psychiatric symptoms or rhabdomyolysis.

## 3 Discussion

In this case, rhabdomyolysis was observed after the patient took a high dose of risperidone. Both CK and myoglobin levels were elevated, and the patient experienced generalized myalgia and fatigue. Rhabdomyolysis is defined as muscle symptoms associated with a CK level elevation greater than 10 times the upper limit of normal and is usually associated with brown urine and the presence of myoglobin in urine [9]; on the basis of these criteria, the patient was diagnosed with rhabdomyolysis. No mechanical trauma or muscle ischemia was observed. Furthermore, since no endocrine disorders, hereditary disorders, extreme exercise, prolonged immobility, or extreme body temperatures were observed, the cause of rhabdomyolysis was most likely drug related, though the possibility that prolonged immobility (approximately 10 h) contributed to muscle injury cannot be excluded.

Elevated creatine kinase levels and rhabdomyolysis are commonly observed in cases of neuroleptic malignant syndrome (NMS), which is a serious adverse reaction to antipsychotic treatment. NMS is usually diagnosed based on clinical findings, including recent antipsychotic use, severe muscle rigidity, a fever greater than 38°C, altered consciousness, autonomic instability, and markedly elevated creatine kinase levels—usually exceeding four times the upper normal limit—in the absence of other identifiable causes. In the present case, however, the patient's clinical presentation did not meet the diagnostic criteria for NMS. Therefore, it is believed that the skeletal muscles were destroyed by the high blood levels of risperidone resulting from the intake of a total of 75 mg of the drug. There are several possible mechanisms underlying skeletal muscle destruction by risperidone. It has been hypothesized that atypical antipsychotics may directly or indirectly affect muscle cell function by regulating serotonin (5‐HT) signaling [10], which, in turn, may affect CK levels and contribute to myotoxicity [11]. Risperidone is an atypical antipsychotic with pharmacological properties that include not only dopamine D2 receptor blockade but also blockade of the 5‐HT pathway, specifically the 5‐HT2A receptor [12]. The role of 5‐HR receptors in skeletal muscle is still unknown, but these receptors are expressed in skeletal muscle and are involved in the regulation of glucose uptake and muscle differentiation [13].

Skeletal muscle is a highly metabolically active tissue that is particularly susceptible to pharmacological insults due to the significant energy demands required to maintain sarcolemmal membrane potential, especially during contraction [14]. The myofiber plasma membrane, being excitable, consumes substantial energy, and when damaged, membrane failure permits extracellular calcium influx into the myocyte [1]. Excess intracellular calcium triggers localized hypercontraction and physical disruption of myofibers, leading to focal rather than diffuse necrosis [14]. Although minor myonecrosis can occur routinely with partial regeneration, extensive muscle damage results in significant release of intracellular components, including myoglobin, into the circulation [15]. The systemic presence of myoglobin can lead to its deposition in renal tubules, where the heme moiety exerts direct nephrotoxic effects, potentially causing acute kidney injury [15]. Preclinical studies have further demonstrated that elevated serotonin levels may accumulate in skeletal muscle tissue via passive diffusion, contributing to cellular toxicity, structural damage, and elevated serum creatine kinase (CK) concentrations [16]. In particular, excessive doses of risperidone may exacerbate muscle vulnerability through antagonism of 5‐HT2A receptors, increasing sarcolemmal permeability, calcium influx, and subsequent intracellular calcium overload [17]. This calcium excess activates calcium‐dependent proteases and lipases, initiating proteolysis and lipid degradation, which, combined with mitochondrial calcium overload that impairs oxidative phosphorylation and ATP synthesis, leads to further muscle fiber breakdown [17]. Collectively, these pathophysiological mechanisms underlie the development of rhabdomyolysis observed in patients receiving atypical antipsychotic medications at toxic doses [18].

There have been several case reports of rhabdomyolysis during treatment with risperidone. Interactions of this drug with simvastatin [5], cerivastatin [6], escitalopram [7], and mirtazapine [8] have been reported, but it is not clear which of these drugs is the culprit drug for rhabdomyolysis.

Although rhabdomyolysis is a recognized adverse event with antipsychotics in general, cases associated specifically with risperidone overdose have not been reported to date. In addition, there have been many reports of rhabdomyolysis following the ingestion of large doses of psychotropic drugs, such as caffeine [19], diphenhydramine [20], acetaminophen [21], ibuprofen [22], heroin [23], methadone [24], olanzapine [25, 26, 27], lamotrigine [28, 29], and haloperidol [30, 31]. Therefore, although the pharmacological mechanisms of action may differ, it is necessary to monitor for the possibility of rhabdomyolysis development when a patient is taking any of these drugs, even when taken orally.

In conclusion, this case shows that even with risperidone, which is the most widely used drug in the world, serious muscle damage may occur when it is taken orally. This case highlights the importance of monitoring for signs of rhabdomyolysis in patients who have overdosed on a psychiatric drug.

## Author Contributions

J.T., K.K., and R.I. were involved in the clinical investigations. J.T. and N.Y.‐F. wrote the manuscript. J.T., K.K., R.I., Y.K., N.S., and N.Y.‐F. were involved in the literature review. All the authors have read and approved the final manuscript.

## Ethics Statement

Approval of the Research Protocol by an Institutional Review Board: The ethics committee of the School of Medicine at Dokkyo Medical University determined that there was no need to review this case.

## Consent

Written informed consent was obtained from the patient's family for the publication of this case report.

## Conflicts of Interest

The authors declare that they have no competing interests to report. Norio Yasui‐Furukori is an editorial board member of *Neuropsychopharmacology Reports* and a coauthor of this article. To minimize bias, they were excluded from all editorial decision‐making related to the acceptance of this article for publication.

## Data Availability

The authors have nothing to report.
